# DA-IRRK: Data-Adaptive Iteratively Reweighted Robust Kernel-Based Approach for Back-End Optimization in Visual SLAM

**DOI:** 10.3390/s25082529

**Published:** 2025-04-17

**Authors:** Zhimin Hu, Lan Cheng, Jiangxia Wei, Xinying Xu, Zhe Zhang, Gaowei Yan

**Affiliations:** Electrical and Power Engineering, Yingxi Campus, Taiyuan University of Technology, No. 79 Yingze West Street, Wanbailin District, Taiyuan 030024, China; 15392639724@163.com (Z.H.); weijiangxia0405@link.tyut.edu.cn (J.W.); xuxinying@tyut.edu.cn (X.X.); zhangzhe@tyut.edu.cn (Z.Z.); yangaowei@tyut.edu.cn (G.Y.)

**Keywords:** visual SLAM, back-end optimization, median absolute deviation, data-adaptive, iteratively reweighted robust kernel

## Abstract

Back-end optimization is a key process to eliminate the cumulative error in Visual Simultaneous Localization and Mapping (VSLAM). Existing VSLAM frameworks often use kernel function-based back-end optimization methods. However, these methods typically rely on fixed kernel parameters based on the chi-square test, assuming Gaussian-distributed reprojection errors. In practice, though, reprojection errors are not always Gaussian, which can reduce robustness and accuracy. Therefore, we propose a data-adaptive iteratively reweighted robust kernel (DA-IRRK) approach, which combines median absolute deviation (MAD) with iteratively reweighted strategies. The robustness parameters are adaptively adjusted according to the MAD of reprojection errors, and the Huber kernel function is used to demonstrate the implementation of the back-end optimization process. The method is compared with other robust function-based approaches via the EuRoC dataset and the KITTI dataset, showing adaptability across different VSLAM frameworks and demonstrating significant improvements in trajectory accuracy on the vast majority of dataset sequences. The statistical analysis of the results from the perspective of reprojection error indicates DA-IRRK can tackle non-Gaussian noises better than the compared methods.

## 1. Introduction

The evolution of Visual Simultaneous Localization and Mapping (VSLAM) technology has been driven primarily by its widespread adoption in mobile robotics, autonomous navigation systems, and immersive reality applications [1]. However, real-world environmental complexities continue to challenge existing VSLAM approaches [2]. Key challenges include maintaining accuracy and robustness when processing noisy, incomplete data containing outliers.

Fundamentally, VSLAM solves state estimation by determining camera poses and reconstructing 3D environments from visual data. The technique’s dependence on visual features makes it vulnerable to motion artifacts and texture ambiguities [3]. Mismatched feature correspondences often generate outliers that compromise system accuracy [4].

Back-end optimization plays a pivotal role in refining VSLAM’s mapping precision. Pioneer VSLAM implementations predominantly utilized filter-based back-end optimizations. Such filters often fail to achieve global consistency while exhibiting outlier sensitivity [5]. Modern VSLAM systems like ORB-SLAM2/3 [3,6] predominantly leverage nonlinear optimization.These approaches formulate pose estimation and mapping as optimization tasks, minimizing cost functions through specialized algorithms.

Contemporary frameworks like ORB-SLAM3 and CCM-SLAM [7] enhance robustness through kernel-function-based optimization. Conventional implementations typically employ fixed-parameter kernel functions. Diverse kernel functions (Huber, Cauchy, Tukey, Geman-McClure) [8,9,10,11] have emerged for different scenarios. Kernel selection remains challenging, given environmental variability and limited function choices. Parameter estimation is further complicated by interdependent shape and scaling parameters. Moreover, few enhanced methods have been validated in actual VSLAM implementations.

To address the above issues, various recent studies have considered data adaptation methods. Ref. [12] proposed a generalized robust loss function for machine learning only. Ref. [13] proposed a new data-driven criterion to solve the robustness parameter of the Huber estimator, to achieve an adaptive Huber estimator and [14] applied the iteratively reweighted strategy to the adaptive robust parameter estimation to optimize the linear regression model problem.

Inspired by [13,14], we propose an iteratively reweighted robust kernel function method based on data adaptation to solve the VSLAM back-end optimization problem. Different from previous methods, the proposed method only needs to estimate the robustness parameter of the kernel function according to the reprojection error data in VSLAM, which can avoid the coupling problem of multi-parameter estimation. Furthermore, the robustness parameter can be adaptively adjusted according to the environmental changes to achieve better optimization results for different scenes. Our main contributions are as follows:

(1) A data-adaptive iteratively reweighted robust kernel-based (DA-IRRK) method is proposed for back-end optimization in VSLAM. In this method, a kernel function is adopted as the objective function for the back-end optimization problem. The robustness parameter in the kernel function is adaptively updated according to the reprojection error, which is reflected through the median absolute deviation (MAD). The proposed method brings out robustness for different scenarios. In addition, the formulated back-end optimization problem is solved iteratively through a reweighted updating process.
(2) The proposed method is implemented in different VSLAM frameworks, including ORB-SLAM3, JORB-SLAM, and CCM-SLAM, to demonstrate its effectiveness in visual-only SLAM, multi-sensor fusion SLAM, and collaborative VSLAM. The proposed method is tested on both indoor and outdoor datasets and compared with other robust kernel methods as well as the state-of-the-art MCMCC method.
(3) The performance difference between the proposed method and other methods is analyzed from the perspective of reprojection error statistics, which provides insights into the VSLAM back-end problem in the context of adaptivity.

The remainder of the paper is organized as follows. Section 2 reviews related work on the VSLAM framework and back-end optimization. Section 3 describes the back-end optimization problem in VSLAM, presents the proposed DA-IRRK method, and takes the Huber kernel as an example to demonstrate the implementation of the DA-IRRK method. Section 4 shows experimental validation for three different modes of visual-only SLAM, multi-sensor fusion VSLAM, and collaborative VSLAM in different VSLAM frameworks, including ORB-SLAM3, JORB-SLAM, and CCM-SLAM. Section 5 draws conclusions and outlines future work.

## 2. Related Work

In this section, we focus on back-end optimization methods for VSLAM. Since different VSLAM frameworks use different back-end optimization methods, we first analyze different VSLAM frameworks and then investigate the existing back-end optimization methods. Finally, we discuss data-driven adaptive-based optimization methods.

### 2.1. VSLAM Frameworks

VSLAM frameworks can be classified into three categories: single-robot VSLAM, multi-robot VSLAM, and CNN-based VSLAM [15]. Single-robot VSLAM systems, such as ORB-SLAM3 [6], are extensively studied for their fast speed and promising accuracy in various scenarios. Multi-robot VSLAM frameworks, such as CCM-SLAM [7] and JORB-SLAM [16], use multiple robots to map complex environments and can address the problems of high individual cost and centralized computation faced by single-robot SLAM systems [17]. Advanced CNN-based VSLAM frameworks [18,19] have also been explored due to the performance improvement by Convolutional Neural Networks (CNN). However, CNN-based VSLAMs, often time-consuming and scenario-specific, need further performance enhancements for real-time applications. In this study, we adopt the single-robot SLAM framework ORB-SLAM3 and the multi-robot VSLAM frameworks CCM-SLAM and JORB-SLAM to demonstrate and verify the proposed method.

### 2.2. Back-End Optimization

Back-end optimization refines the preliminary estimates from the front end in VSLAM. Filter-based [5] methods and nonlinear optimization methods [20] are currently the most popular for back-end optimization. In early VSLAM implementations, filter-based methods, such as the Extended Kalman Filter (EKF) method, were prevalent. However, the EKF lacks an outlier elimination mechanism [21,22], and even a few outliers may significantly degrade the optimization performance. Furthermore, in large-scale VSLAM, the growing number of frames and map points slows the optimization process as it requires dynamic updates of both the mean and the variance, along with the state size. On the other hand, nonlinear optimization methods, such as bundle adjustment, which formulate the state estimation of the camera pose and map points as an optimization problem, dominate the mainstream SLAM frameworks (such as PTAM [23], ORB-SLAM3, and DSO [24]). Notably, most modern SLAM systems, including ORB-SLAM3, rely on general-purpose optimization back-ends like g2o [25] rather than implementing their own optimization modules. These frameworks usually adopt Gauss–Newton [26] or Levenberg–Marquardt (L–M) algorithms [27] to solve the optimization problem.

### 2.3. Robust Kernel Functions

Most nonlinear optimization methods aim to minimize the sum of squared error to counter mismatches. If the data samples are outliers, this approach tends to fail to identify incorrect data and cause estimation errors. Consequently, using robust kernel functions as the objective function has become a common practice to prevent errors on one edge from overshadowing others [28]. Refs. [4,29] applied these kernel functions to various estimation problems in computer vision and robotics. Ref. [30] analyzed popular robust kernel functions for alignment problems, offering suggestions for kernel function selection based on the application scenarios.

However, the abovementioned methods manually select different robust kernel functions depending on the situation, lacking flexibility and robustness for new scenes. Ref. [31] introduced a generalized adaptive robust kernel function, which makes the above robust kernel functions a special case of the generalized robust kernel function. By setting the shape parameter differently, the generalized adaptive robust kernel function can automatically select a suitable robust kernel function according to different situations. Ref. [32] improved the generalized robust kernel family based on [31] by adapting robust kernel shapes through the probability distribution of the generalized loss function and applying the improved method to nonlinear optimization problems. Ref. [33] associated the M-estimator with an elliptic probability distribution. They estimate hyper-parameters for each kernel type based on the residual distribution and perform model comparisons to determine the best kernel for the situation at hand. All of the above adaptive methods focus on estimating shape parameters and then finding a suitable robust kernel function.

### 2.4. Adaptive Methods

With the popularity of data-driven approaches, various recent works have reconsidered data-adaptive methods, giving rise to two fundamental parameter optimization paradigms: adaptive methods and self-learning mechanisms [34]. Adaptive methods (e.g., the M-estimator parameter adjustment proposed by [35]) primarily tune model parameters by analyzing real-time data distribution characteristics (such as reprojection errors), with [35] demonstrating that such adaptive methods significantly enhance robustness compared with static M-estimators across different data distributions. In contrast, self-learning mechanisms (as seen in recent path planning research) achieve continuous system self-optimization through built-in feedback loops.

Early ideas for adaptively choosing the tuning parameters of M-estimators are presented in [35] based on different data distributions. Recent studies by [13,36] have further validated the necessity of data-dependent adjustment for Huber estimator parameters to effectively suppress outliers, showing these adaptive parameters can be customized according to variations in sample size, dimensionality, and noise characteristics. Ref. [14] advanced this direction by combining iteratively reweighted least squares with adaptive algorithms for parameter estimation, successfully applying this methodology to linear regression model optimization problems.

In this paper, we propose a data-adaptive iteratively reweighted robust kernel-based (DA-IRRK) approach by drawing on the data-driven adaptive robust Huber kernel function method and iteratively reweighted strategy in [14,35], in which the robustness parameter of the robust kernel function is adaptively changed based on the distribution of reprojection error in the real environment, rather than a fixed robustness parameter in all cases. The proposed method can be implemented in the back end of any VSLAM framework.

## 3. Back-End Optimization Based on DA-IRRK

**Figure 1:**
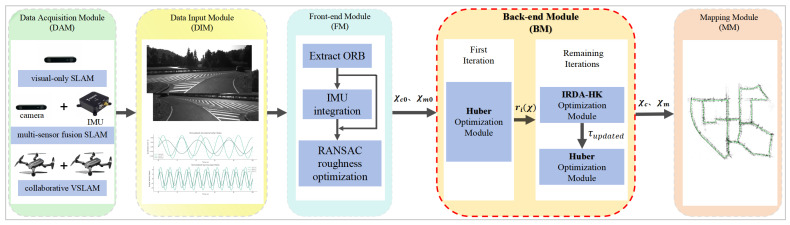
General framework. The framework illustrates three forms of VSLAM: single-robot VSLAM, multi-sensor fusion VSLAM, and multi-robot VSLAM. The data input module transmits data to the front-end module for feature extraction, IMU data integration, and RANSAC optimization. The back-end module performs iterative optimization. Optimized camera positions and map points serve as inputs for the mapping module, facilitating mapping.

Figure 1 illustrates the general VSLAM framework that the proposed back-end optimization method applies to. We focus on the VSLAM back-end module. We elaborated the proposed method and its implementation following the preliminary work related to back-end optimization.

### 3.1. Back-End Optimization

**Figure 2:**
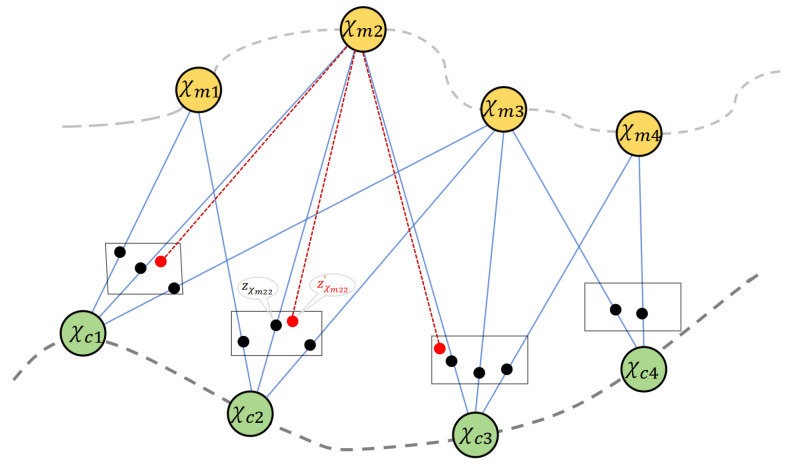
Illustration of the reprojection process. $\chi_{mj}$ represents the 3D map point in global reference system, and $\chi_{ci}$ denotes the camera position. The black box signifies the pixel coordinate system, with the black point indicating the observation and the red point representing the 2D reprojection. The blue solid line depicts the projection process, while the red dashed line illustrates the reprojection process. Additionally, two gray dashed lines trace both the map point trajectory and camera trajectory, respectively.

State estimation in VSLAM involves determining the unknown parameters of the motion model based on noisy observations $z_{ij}$, which include camera position $\chi_{c}$ and map point $\chi_{m}$, collectively termed χ. The state estimation problem can be viewed as a nonlinear least squares (LS) optimization problem in the back end. While Equation (1) presents the general formulation, practical implementations in frameworks like ORB-SLAM3 using g2o solve a modified version that accounts for additional engineering considerations [25]. Back-end optimization consists of both local and global optimization: local optimization refines the pose and map points of a few keyframes locally, while global optimization optimizes all keyframes after loop closure detection. The optimization problem is typically solved by minimizing the objective function of reprojection error, expressed as follows:

(1) $$X^{*}=\mathrm{argmin}L\left(\chi \right)=\mathrm{argmin}\frac{1}{2}\sum_{i=1}^{N}\sum_{j=1}^{M}\omega_{ij}{∥e_{ij}\left(\chi \right)∥}^{2}$$

where *i* varies from 1 to *N*, and *N* is the number of frames; *j* varies from 1 to *M*, and *M* is the number of map points that can be seen from the position of the *i*-th frame. The reprojection error, denoted as $e_{ij}\left(\chi \right)=z_{ij}-f_{ij}\left(\chi \right)$ refers to the discrepancy between the camera observation $z_{ij}$ and the reprojected 3D map point $\chi_{mj}$ onto the *i*-th 2D frame using the projection function $f_{ij}\left(\cdot \right)$. The weight $\omega_{ij}$ represents the information matrix (inverse covariance $\Sigma_{ij}^{-1}$), encoding the uncertainty of observation $z_{ij}$ under the Gaussian assumption $z_{ij}∼N\left(f_{ij}\left(\chi \right),\Sigma_{ij}\right)$. The observation $z_{ij}$ represents $\chi_{mj}$ in the *i*-th frame, typically in pixel coordinates. The importance of the *i*-th reprojection error is represented by its weight $\omega_{ij}$. Figure 2 illustrates the reprojection process in VSLAM. While $X^{*}$ is statistically optimal when the error of the observation $z_{ij}$ in the *i*-th frame follows a Gaussian distribution, it may deviate from this optimal solution in cases involving non-Gaussian noises [11].

When $e_{ij}\left(\chi \right)$ follows a Gaussian distribution, the weighted LS problem shown in Equation (1) can be directly solved by the Gauss–Newton algorithm or Levenberg–Marquardt (L–M) algorithm. However, due to the non-convex characteristics of the image and the influence of environmental noises, $e_{ij}\left(\chi \right)$ typically follows a sub-Gaussian distribution or non-Gaussian distribution. The estimation results obtained using only the Gauss–Newton algorithm or L–M algorithm often deviate from the true value, leading to a decrease in localization and mapping accuracy in VSLAM.

### 3.2. DA-IRRK

In the field of VSLAM, heavy-tailed distribution data affected by sub-Gaussian or non-Gaussian noises are often observed in both datasets and real scene experiments, leading to results with large errors [37]. Robust kernel functions with specific robustness parameters are widely used in the field of VSLAM to solve the back-end optimization problem and improve mapping accuracy, as the robustness parameter can balance accuracy and robustness. According to [38], the robustness parameter should be adapted to the sample size, dimensionality, noise variance, and confidence level of the experimental data. While Equation (2) presents a general form of robust kernel formulation, practical implementations like ORB-SLAM3 properly incorporate the information matrix of measurements when applying the Huber kernel in their g2o-based optimization back-end. In VSLAM, Equation (2) shows the objective function based on the robust kernel function, where l(·) represents the robust loss or kernel.

(2) $$X^{*}=\mathrm{argmin}L\left(\chi \right)=\mathrm{argmin}\sum_{i=1}^{N}\sum_{j=1}^{M}l\left(e_{ij}\left(\chi \right)\right)$$

In previous studies, the robustness parameter is typically set to a fixed value to adapt to specific scene noises. However, this approach requires re-determining the robustness parameter whenever the scene changes, which results in the designed back-end optimization algorithm having poor robustness and low mapping accuracy in practical applications. To address this issue, an adaptive back-end optimization algorithm is proposed that can adaptively adjust the robustness parameters according to different scenarios, thereby improving the robustness of the back-end and enhancing the mapping accuracy.

The Huber kernel function is a popular robust kernel function in VSLAM, which is widely used in mature VSLAM back-end optimization modules such as ORB-SLAM2 and ORB-SLAM3. Therefore, in the following algorithm presentation, the proposed method is demonstrated using the Huber kernel function as an example. The Huber kernel function and its corresponding first-order derivatives are presented in Equations (3) and (4), where τ represents the robustness parameter and *e* is the error.

(3) $$l_{\mathrm{Huber}\;}\left(e\right)=\left\{\begin{array}{c} \frac{1}{2}e^{2},\;\;\;if\left|e\right|\leq \tau \\ \tau |e|-\frac{1}{2}\tau^{2},\;if\left|e\right|>\tau \end{array}\right.$$

(4) $$\varphi_{\mathrm{Huber}\;}\left(e\right)=\left\{\begin{array}{c} e,\;\;\;\;if|e|\leq \tau \\ sgn(e)\tau ,\;if|e|>\tau \end{array}\right.$$

Equations (3) and (4) demonstrate that τ plays a crucial role in determining the weight of the errors in the optimization process. When *e* is less than or equal to τ, the back-end optimization problem based on the Huber kernel function can be regarded as an LS problem, corresponding to the case where $\omega_{ij}=1$ in Equation (1). On the other hand, when *e* exceeds τ, the influence of error samples on the optimization process is significantly suppressed. A comprehensive analysis provided by [20] concludes that the choice of τ determines the robustness and sensitivity of the back-end optimization algorithm. Therefore, we propose an adaptive tuning approach for τ in our back-end optimization algorithm based on the Huber kernel function. The aim is to solve the optimization problem shown in Equation (2) using an iteratively reweighted strategy.

To solve Equation (2), we utilize the adaptive method proposed by [39] and integrate it with existing back-end optimization algorithms, such as the L–M algorithm, which can be implemented in the following five steps:

(1) Calculate the current reprojection error based on the camera pose and map points at the front end;
(2) Determine the adaptive threshold σ according to Equation (6) after computing the MAD of reprojection errors using Equation (5), where $median\left(x\right)$ represents the median of a set of data and $x_{i}$ represents the *i*-th sample in the set. The MAD strategy is preferred over standard deviation due to its higher breakdown point (50% vs. 0%) for outlier resistance [40], which is crucial for handling outliers in SLAM. The coefficient 1.4826 in Equation (6) equals $1/\Phi^{-1}\left(0.75\right)$, where $\Phi^{-1}$ is the inverse standard normal CDF. This scaling ensures $\sigma_{MAD}$ matches the standard deviation for normally distributed data, maintaining compatibility with Gaussian kernels [41]. This adaptive mechanism enables automatic adjustment of the robustness parameters based on real-time sensor data characteristics, representing a key advantage over traditional fixed-parameter methods like MCMCC. These computations are performed in the tangent space of the manifold, which provides a vector space approximation for the nonlinear optimization problem while maintaining the geometric properties of the original space. It is worth noting that the MAD strategy may not be sufficiently robust for data distributions containing a larger number of large outliers;
  (5) $$\mathrm{MAD}\left(x\right)=median\left(|x_{i}-\right.median\left.(x)|\right)$$
  (6) $$\sigma_{MAD}\left(e_{ij}\left(\chi \right)\right)=\frac{\mathrm{MAD}\left(e_{ij}\left(\chi \right)\right)}{\varphi^{-1}\left(0.75\right)}$$
(3) Calculate the robustness parameter in the Huber kernel function using Equation (7), where *c* represents the scaling factor. The scale factor $c=\sqrt{5.99}$ corresponds to 95% $\chi^{2}$ confidence. T is the vector form of τ;
  (7) $$T=c\sigma_{MAD}\left(e_{ij}\left(\chi \right)\right)$$
(4) Solve the objective function iteratively through a reweighted updating process;
(5) Utilize the L–M algorithm to update the camera pose and map points.

After completing the previous three steps, it is necessary to find the derivative of L(χ) before performing the subsequent steps, which are represented as Equation (8), and the iteratively reweighted strategy is used for further simplification, as in Equation (9). The iteratively reweighted strategy has also been used in [42] to achieve reweighting in the optimization equations, and experimental results show its effectiveness.

(8) $$\frac{\partial L(\chi )}{\partial \chi }=\sum_{i=1}^{N}\sum_{j=1}^{M}\frac{dl\left(e_{ij}\left(\chi \right)\right)}{de_{ij}\left(\chi \right)}\frac{\partial e_{ij}\left(\chi \right)}{\partial \chi }$$

(9) $$\frac{\partial L(\chi )}{\partial \chi }=\sum_{i=1}^{N}\sum_{j=1}^{M}\frac{dl\left(e_{ij}\left(\chi \right)\right)}{de_{ij}\left(\chi \right)}\frac{\partial e_{ij}\left(\chi \right)}{\partial \chi }=\sum_{i=1}^{N}\sum_{j=1}^{M}\omega_{ij}e_{ij}\left(\chi \right)\frac{\partial e_{ij}\left(\chi \right)}{\partial \chi }=\sum_{i=1}^{N}\sum_{j=1}^{M}\omega_{ij}\left(\chi \right)e_{ij}\left(\chi \right)\frac{\partial f_{ij}\left(\chi \right)}{\partial \chi }=0$$

where the first-order derivative of $l\left(e_{ij}\left(\chi \right)\right)$ is defined as $l^{\prime }\left(e_{ij}\left(\chi \right)\right)=\frac{dl\left(e_{ij}\left(\chi \right)\right)}{de_{ij}\left(\chi \right))}$, $l^{\prime }\left(e_{ij}\left(\chi \right)\right)$ represents the influence function and $\omega_{ij}\left(\chi \right)=\frac{l^{\prime }\left(e_{ij}\left(\chi \right)\right)}{e_{ij}\left(\chi \right)}$ is the weight function. After simplifying Equation (2) using the iteratively reweighted strategy described above, the optimization of Equation (2) can be further achieved by the L–M algorithm.

In the L–M algorithm, the projection equation $f_{ij}\left(\chi \right)$ is firstly Taylor expanded (Equation (10)), then Equations (11) and (12) are solved sequentially, and finally, the matrix form of the solution to Equation (12) is expressed as Equation (13), which is the update vector Δχ of the L–M algorithm after adding the damping coefficient λ (Equation (14)).

(10) $$f_{ij}\left(\chi \right)\approx f_{ij}\left(\chi^{k}\right)+\sum \frac{\partial f_{ij}\left(\chi^{k}\right)}{\partial \chi }\left(\chi -\chi^{k}\right)=f_{ij}\left(\chi^{k}\right)+\sum J\cdot \Delta \chi$$

(11) $$e_{ij}\left(\chi \right)=z_{ij}-f_{ij}\left(\chi \right)=z_{ij}-f_{ij}\left(\chi^{k}\right)+f_{ij}\left(\chi^{k}\right)-f_{ij}\left(\chi \right)\approx \Delta e_{ij}-\sum J\cdot \Delta \chi$$

(12) $$\frac{\partial L(\Delta \chi )}{\partial \Delta \chi }=-\sum_{i=1}^{N}\sum_{j=1}^{M}\omega_{ij}\left(\Delta \chi \right)\cdot \left(\Delta e_{ij}-\sum J\cdot \Delta \chi \right)\cdot \frac{\partial f_{ij}\left(\Delta \chi \right)}{\partial \Delta \chi }=\sum_{i=1}^{N}\sum_{j=1}^{M}\omega_{ij}\left(\Delta \chi \right)\cdot \left(\Delta e_{ij}-\sum J\cdot \Delta \chi \right)\cdot J=0$$

(13) $$\Delta \chi ={\left(J^{T}WJ\right)}^{-1}J^{T}W\Delta E$$

(14) $$\Delta \chi ={\left(J^{T}WJ+\lambda \cdot \mathrm{diag}\left(J^{T}WJ\right)\right)}^{-1}J^{T}W\Delta E$$

where W is treated as a diagonal matrix.

During the iteration process, when λ approaches zero, it signifies that the L–M algorithm behaves similarly to the Gauss–Newton method. Conversely, a high damping coefficient aligns the update vector with the direction of the gradient, making the L–M algorithm a compromise between the Gauss–Newton algorithm and gradient descent. The implementation of the proposed method DA-IRRK is summarized in Algorithm 1.

It is worth noting that $\omega_{ij}$ is updated in each iteration, which is also why we name our method an iterative reweighted method. As the iteration carries on, the weight $\omega_{ij}$ reflects the importance of each $e_{ij}$ with better and better accuracy, leading to the approaching of the global solution.
**Algorithm 1** DA-IRRK-based back-end optimization.**Require:** Camera pose $\chi_{c}$ and map point $\chi_{m}$, denoted as χ in the algorithm; 
**Ensure:** Updated model parameters $\chi_{c}$ and $\chi_{m}$;
  1:Initialization: Set the initial camera pose and map point $\chi_{0}$. Define the robustness parameter T of the robust kernel and set the parameter σ of the DA-IRRK method to zero;  2:**for** k=1 to *N* **do**  3:    **if** k=1 **then**  4:        Calculate the $e_{ij}\left(\chi \right)$ using $e_{ij}\left(\chi \right)=z_{ij}-f_{ij}\left(\chi \right)$, update $e_{ij}\left(\chi \right)$ using Equation (2);  5:        Compute σ and T according to Equations (5)–(7);  6:        Define the updated σ as $\sigma_{0}$, which is passed into the DA-IRRK algorithm;  7:    **else**  8:        With the incoming $\sigma_{0}$ and updated $e_{ij}\left(\chi \right)$, compute T of the DA-IRRK method using Equation (7);  9:        Substitute the obtained T into Equations (3) and (4);10:        The objective function of Equation (2) is derived using Equations (8)–(10) to obtain the update quantity Δχ;11:        Equation (13) is deformed into Equation (14) and $\chi_{c}$ and $\chi_{m}$ are updated iteratively using the L–M algorithm;12:    **end if**13:**end for**


## 4. Experiments

We evaluate the proposed DA-IRRK method along with other robust kernel function-based back-end optimization methods on both indoor and outdoor datasets in different VSLAM frameworks, including ORB-SLAM3, CCM-SLAM [7], and JORB-SLAM [16]. The EuRoC dataset is utilized for indoor testing, while the KITTI dataset is used for outdoor validation to assess the accuracy and robustness of the DA-IRRK method. Eigen3 [43], G2O [25], PCL [44], Pangolin [45], and OpenCV [46] are employed for implementing the proposed method. All experiments are conducted on a laptop with an Intel Core i5 12400F CPU and 16 GB of RAM running Ubuntu 18.04. Here, we present the detailed implementation of the DA-IRRK method for visual SLAM back-end optimization, specifically realized within the Edge and Solver modules of the G2O library.

1. Adaptive Edge Module: New EdgeSE3-DAIRRK class implements the following:
  - Dynamic kernel parameter computation (Equations (5) and (6)).
  - Real-time robust kernel selection (Equation (7)).
  - Information matrix weighting mechanism.
2. Solver Module: Enhanced LinearSolverEigen features the following:
  - Optimized sparse matrix storage pattern.
  - Improved marginalization strategy.

### 4.1. Experimental Datasets

The DA-IRRK method was evaluated across two distinct environments: indoor scenarios using the EuRoC dataset [47] and outdoor settings with KITTI [48]. EuRoC captures three environment types: ETH Zurich’s machine halls (MH01-MH05), calibration chambers (V101-V103), and standard rooms (V201-V203). Based on feature availability and UAV flight dynamics, EuRoC sequences are categorized by difficulty:

- Simple: MH01 (80.6 m), MH02 (73.5 m), V101 (58.6 m), V201 (36.5 m).
- Moderate: MH03 (130.9 m), V102 (75.9 m), V202 (83.2 m).
- Challenging: V103 (79.0 m), V203 (86.1 m), MH04 (91.7 m), MH05 (97.6 m).

The KITTI dataset includes urban, residential, rural, and highway scenarios. The primary data acquisition equipment includes a 64-beam Velodyne laser scanner, four cameras, and a GPS/IMU unit. We select 11 sequences (00–10) to verify the proposed method in the outdoor environment. The sequence lengths are as follows: sequence 00: 3724.187 m; sequence 01: 2453.203 m; sequence 02: 5067.233 m; sequence 03: 560.888 m; sequence 04: 393.645 m; sequence 05: 2205.576 m; sequence 06: 1232.876 m; sequence 07: 694.697 m; sequence 08: 3222.795 m; sequence 09: 1705.051 m; sequence 10: 919.518 m.

### 4.2. Experimental Frameworks and Evaluation Benchmarks

#### 4.2.1. Experimental Frameworks

We evaluate the robustness of the proposed method by implementing it in ORB-SLAM3 for single-robot applications and in JORB-SLAM and CCM-SLAM for multi-robot systems.

ORB-SLAM3 is a single-robot VSLAM framework that supports multiple cameras and offers visual, visual-inertial, and multi-map SLAM for various applications. It is regarded as the most advanced VSLAM framework to date and is widely used as a benchmark for both indoor and outdoor testing. Notably, the DA-IRRK method is evaluated in ORB-SLAM3 for both single-robot VSLAM and multi-sensor fusion-based VSLAM, as the framework supports both visual-only and visual-inertial modes.

JORB-SLAM is a feature-based multi-robot VSLAM framework that extends ORB-SLAM2 to multiple agents, focusing on rapid map fusion and improved coverage. The system includes multiple ORB-SLAM2 clients for local mapping and a central server for global map fusion. The DA-IRRK method is tested within this framework, as it enhances robustness by adding additional constraints to the AprilTag.

CCM-SLAM is a centralized collaborative multi-robot VSLAM framework based on ORB-SLAM2 that ensures agent autonomy while fostering cooperation through a central server for map fusion and optimization, maintaining efficiency despite potential data loss or delays. Unlike JORB-SLAM, where each agent runs a complete sub-map, CCM-SLAM is widely used as a comparative collaborative VSLAM framework for multi-agent applications, such as CORB2I-SLAM [49] and COVINS [50]. We implement the DA-IRRK method in each CCM-SLAM agent and verify the performance improvement after modification.

#### 4.2.2. Evaluation Metrics

The Absolute Trajectory Error (ATE) [51] is a widely used metric in VSLAM evaluation, measuring the global consistency of the estimated trajectory by comparing it to the ground truth. The accuracy is expressed through the Root Mean Square Error (RMSE) of the ATE. In this paper, the EVO [52] tool is used to compute ATE, providing an assessment of the trajectory accuracy of the VSLAM system against the ground truth. EVO also offers data analysis and visualization for odometry and SLAM evaluation.

### 4.3. Case Study

#### 4.3.1. Visual-Only SLAM

This section evaluates the proposed method in visual-only SLAM mode using ORB-SLAM3 with a single agent and a visual sensor. We compare it with three robust kernel-based methods (Huber, Tukey, and Cauchy, all using ORB-SLAM3’s default kernel parameters) and the state-of-the-art (SOTA) method, MCMCC [42], to assess the RMSE of ATE across different scenarios using the EuRoC and KITTI datasets. Monocular scale blur causes errors in ORB-SLAM3 in open environments, complicating comparisons. Therefore, we evaluate these methods solely in stereo mode, with results presented in Table 1 and Table 2, where the top performer is bolded and the second-best is underlined.

Table 1 demonstrates that the proposed DA-IRRK method achieves higher accuracy in most indoor sequences compared with other methods, while the MCMCC method outperforms ours on MH03, V103, V201, and V203. Table 2 shows that the proposed DA-IRRK method shows lower accuracy than the comparing methods in sequences 03, 06, 09, and 10 on the KITTI dataset. Meanwhile, the MCMCC method outperforms ours in sequences 03 and 10. The analysis method in Table 2 is the same as that in Table 1.

(a) Adaptive Robustness in DA-IRRK vs. Fixed-Parameter Huber Kernel

To further investigate these findings, we analyze sequences V101 and V203 for the EuRoC dataset and sequences 01 and 09 for the KITTI dataset as examples. As noted in Section 3.2, the DA-IRRK method adaptively adjusts the robustness parameter based on reprojection errors to minimize the impact of outliers on mapping accuracy, employing the MAD strategy for robust estimation of data distributions containing outliers (refer to Equations (5)–(7) in Section 3.2).

**Figure 3:**
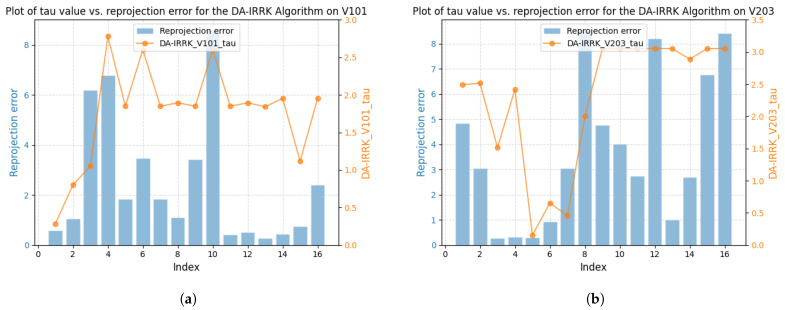
Relationship between reprojection errors and robustness parameter τ for the DA-IRRK method in sequences V101 and V203. (**a**) The left is for V101. (**b**) The right is for V203.

**Figure 4:**
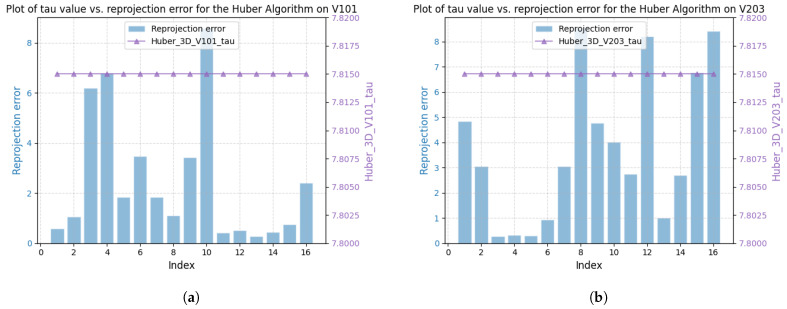
Relationship between reprojection errors and robustness parameter τ for robust Huber kernel method in sequences V101 and V203. (**a**) The left is for V101. (**b**) The right is for V203.

**Figure 5:**
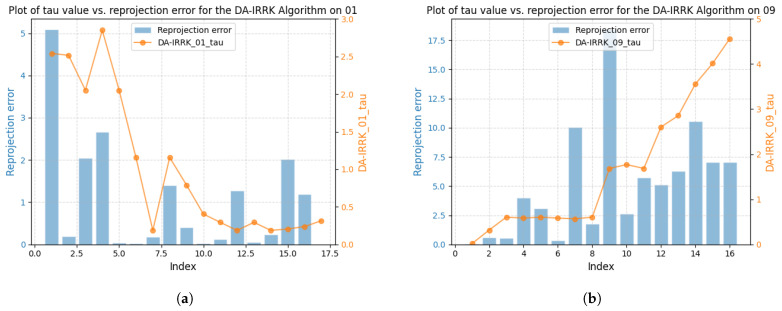
Relationship between reprojection errors and robustness parameter τ for the DA-IRRK method in sequences 01 and 09. (**a**) The left is for 01. (**b**) The right is for 09.

**Figure 6:**
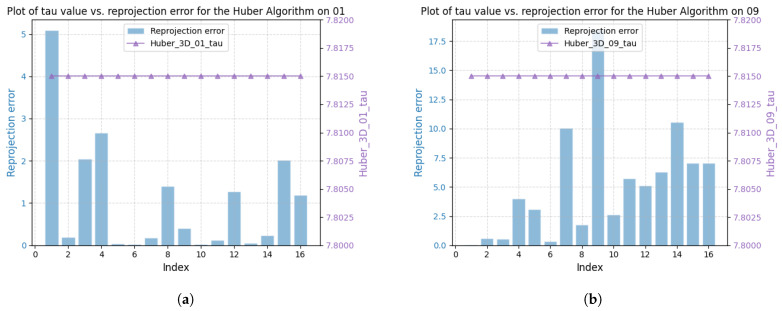
Relationship between reprojection errors and robustness parameter τ for the robust Huber kernel method in sequences 01 and 09. (**a**) The left is for 01. (**b**) The right is for 09.

Figure 3 shows the correlation between reprojection error and robustness parameter for sequences V101 and V203 using the DA-IRRK method, while Figure 4 presents the same for the robust Huber kernel method. The Huber kernel’s robustness parameter is fixed at 5.99 for monoculars and 7.81 for stereo cameras, calculated using the chi-square test [41]. Unlike the Huber kernel, DA-IRRK’s parameter τ varies with reprojection errors, reflecting its data-dependent adaptation. However, the MAD-based estimation in DA-IRRK exhibits sensitivity to extreme outliers, leading to suboptimal performance in sequences like V203 with pronounced non-Gaussian noise (e.g., due to motion blur). Similarly, Figure 5 and Figure 6 show how robustness parameters and reprojection errors vary when applying DA-IRRK and the robust Huber kernel methods to ORB-SLAM3 on sequences 01 and 09. While DA-IRRK’s parameter varies with error, the Huber kernel’s remains constant. Sequence 09 exhibits larger reprojection errors, corresponding to higher robustness parameters. However, as τ is derived from the median error, its magnitude does not strictly correlate with error size—a limitation that occasionally compromises accuracy.

(b) Noise Distribution Analysis and MAD Strategy Efficacy

**Figure 7:**
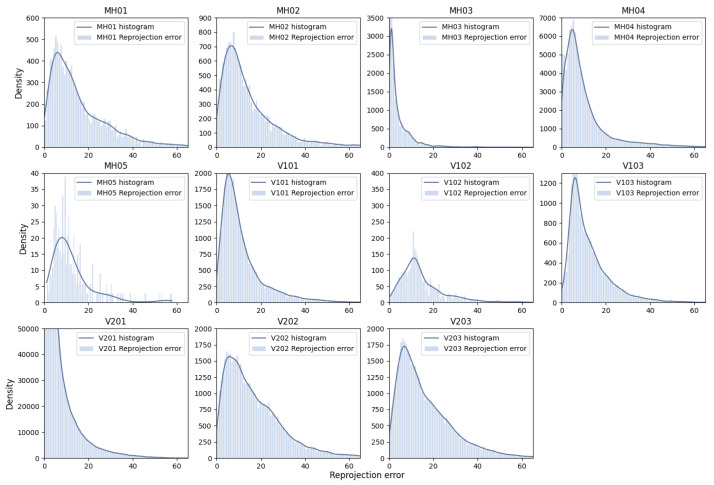
Reprojection error distribution of global keyframes across sequences MH01-V203 in the EuRoC dataset.

**Figure 8:**
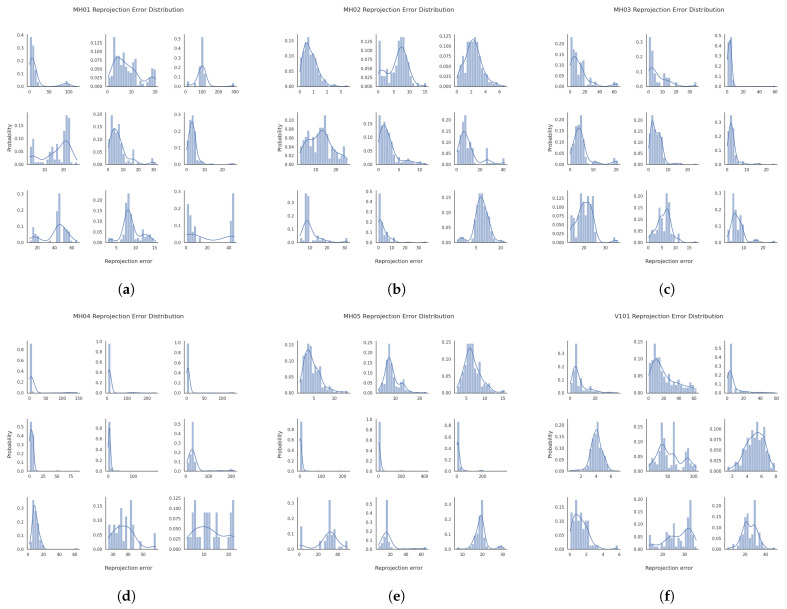

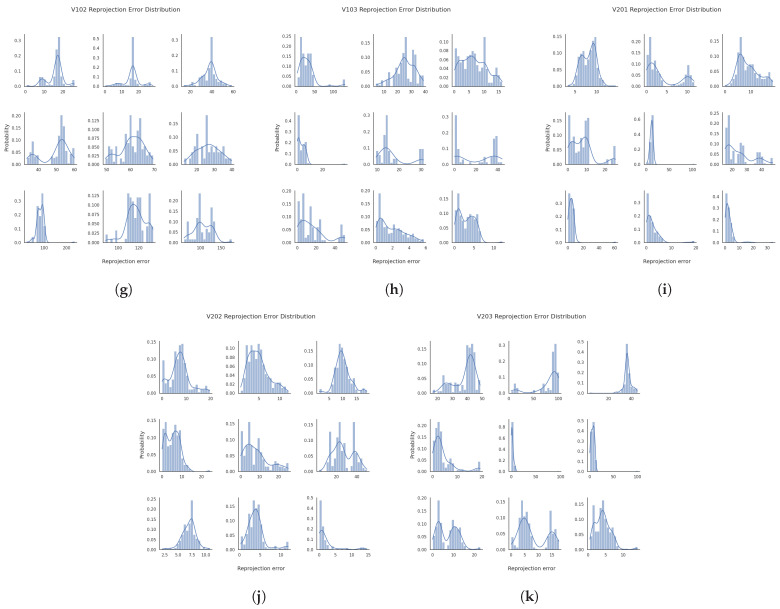
Reprojection error distribution plots of localized keyframes at three different timesteps for sequences MH01-V203 in the EuRoC dataset. The three horizontal subplots per sequence represent one moment. (**a**–**k**) correspond to sequences MH01-V203, respectively.

We assess the impact of the MAD strategy by visualizing the reprojection error distributions for both global and local keyframes in individual sequences from the EuRoC dataset, as shown in Figure 7 and Figure 8. The reprojection error distributions of global keyframes in each sequence show a non-Gaussian pattern. In contrast, local keyframes (Figure 8)—sampled at critical phases (initialization, motion bursts, and stable tracking)—reveal sub-Gaussian distributions for sequences MH01, MH02, MH04, MH05, V101, V102, and V202, while others (e.g., MH03, V201, V203) retain non-Gaussian traits. The MAD-based DA-IRRK method effectively suppresses sub-Gaussian heavy-tailed outliers, achieving superior accuracy in 7 of 11 sequences (Table 1). However, its performance lags behind MCMCC for non-Gaussian outliers (e.g., V203), highlighting a limitation in handling extreme noise. This instability warrants future improvement, as noted in Section 5. Specifically, Figure 7 shows that reprojection errors in sequence V101 are mainly concentrated in the range of 0 to 20, with fewer errors between 20 and 60. In contrast, for sequence V203, errors are concentrated in the 0 to 20 range, but more errors fall between 20 and 60, impacting mapping accuracy. It is important to note that the results presented are snapshots selected from local optimal optimizations at random instances. Reprojection error distributions vary across different instances.

**Figure 9:**
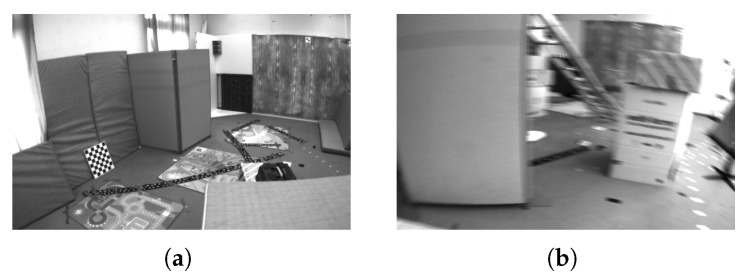
Scenes from EuRoC dataset. (**a**) The left is from V101. (**b**) The right is from V203.

To investigate the root reason for varying reprojection error distributions, we analyze the scenes of sequences V101 and V203. In Figure 9, sequence V101 features brighter scenes and sequence V203 displays blurred scenes caused by fast-moving UAVs. V101 is captured when the UAV flies slowly, resulting in clear scene graphs. On the other hand, sequence V203 is recorded when the UAV is fast-moving, leading to a blurred texture in the scene graph [47]. As a consequence, the front-end feature extraction and matching process of VSLAM produces more mismatches due to the blurred texture, thereby increasing both the number and magnitude of reprojection error outliers.

**Figure 10:**
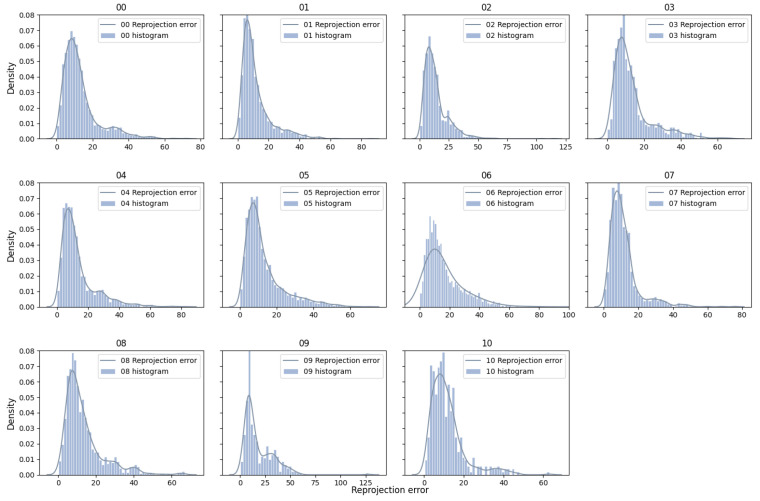
Reprojection error distribution of global keyframes across sequences 00–10 in the KITTI dataset.

**Figure 11:**
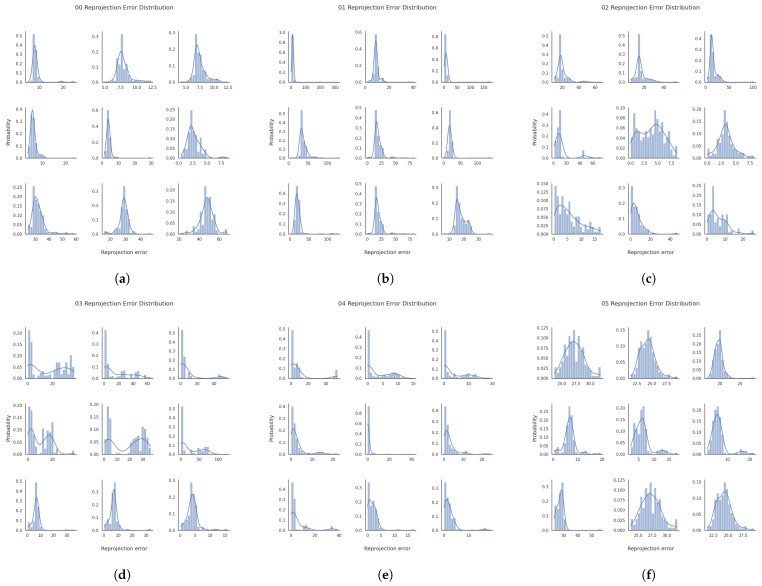

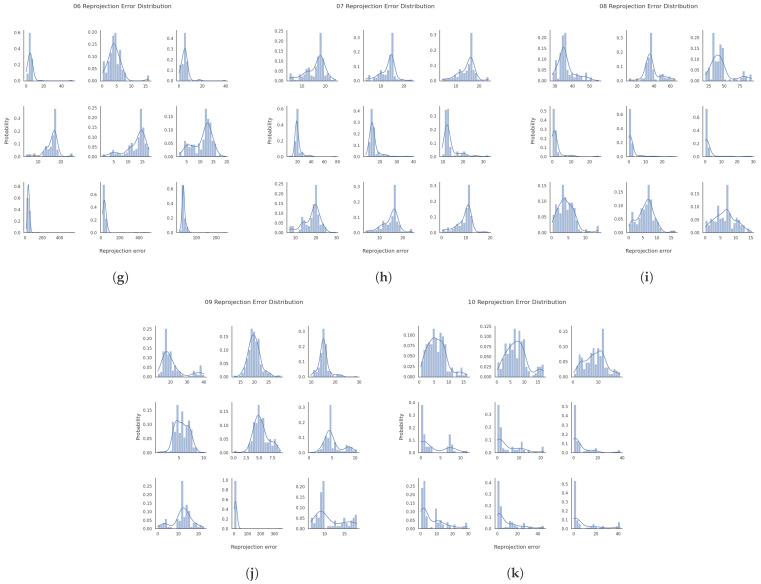
Reprojection error distribution plots of localized keyframes at three different timesteps for sequences 00–10 in the KITTI dataset. The three horizontal subplots per sequence represent one moment. (**a**–**k**) correspond to sequences 00–10, respectively.

The reprojection error distributions of individual sequences from the KITTI dataset in global and local keyframes are shown in Figure 10 and Figure 11. The results indicate that reprojection errors of global keyframes tend to be non-Gaussian distributed, while those of local keyframes are prone to be sub-Gaussian distributed on sequences 00, 01, 02, 04, 05, 07, and 08, whereas on the other sequences, the reprojection errors tend to be non-Gaussian distributed, both in the global keyframes and the local keyframes. Combined with Table 2, we can see that the MCMCC method proves effective at suppressing outliers of non-Gaussian type with heavy-tailed distribution, leading to higher mapping accuracy for sequences 03 and 10.

Specifically, Figure 11 shows that most reprojection errors of sequence 01 are concentrated within the range of 0 to 20, with only a few error samples in the range of 20 to 60. In contrast, reprojection errors of sequence 09 reach a maximum of 125, and a large number of reprojection errors lie in the range of 25 to 75. The average error is larger than that on the other seven sequences, causing the degradation of the mapping accuracy.

**Figure 12:**
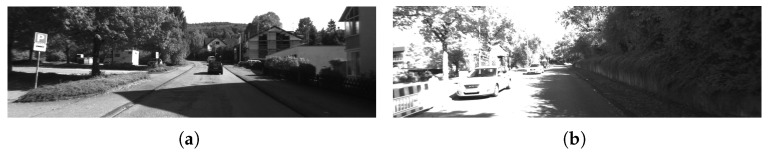
Scenes from EuRoC dataset. (**a**) The left is from V101. (**b**) The right is from V203.

Figure 12 shows that sequence 01 exhibits clearer and more static features with minimal lighting variations, while sequence 09 contains dynamic elements (e.g., a moving car) and displays obvious lighting variations. For sequence 09, the effects of lighting and dynamic features result in more and larger reprojection error outliers.

Based on the above analysis, the results on the KITTI dataset coincide with those on the EuRoC dataset, both demonstrating the effectiveness of the proposed DA-IRRK method for single-robot VSLAM applications with the visual-only case.

#### 4.3.2. Multi-Sensor Fusion VSLAM

To test the proposed DA-IRRK method in a multi-sensor fusion VSLAM mode, we also consider using ORB-SLAM3 in stereo visual-inertial mode. We switch from the visual-only mode to the visual-inertial mode in ORB-SLAM3. In this experiment, we specifically integrate the DA-IRRK method into the g2o optimization library while keeping the IMU-related components unchanged in the backend processing pipeline. Evaluation of the EuRoC dataset is assessed using the RMSE of ATE. Since [42] did not test the proposed MCMCC method in visual-inertial mode, we only provide the results of the proposed DA-IRRK method and the robust Huber kernel method to avoid redundancy since Huber is the most widely used compared with other kernels, and it is only inferior to the proposed method and MCMCC according to Table 1 and Table 2. Detailed results are presented in Table 3, where the top performer is bolded. The third-row Impr_huber in Table 3 quantifies the improvement of the proposed method compared with the Huber function-based method.

Table 3 shows that mapping accuracy has been improved on 9 out of 11 sequences, with 6 sequences indicating an improvement of more than 10%. The highest accuracy improvement is seen at 26.29%. Only the accuracy of mapping on sequences V201 and V203 decreases but by less than 9.5%. This serves as evidence of the effectiveness of the DA-IRRK method in visual-inertial mode.

**Figure 13:**
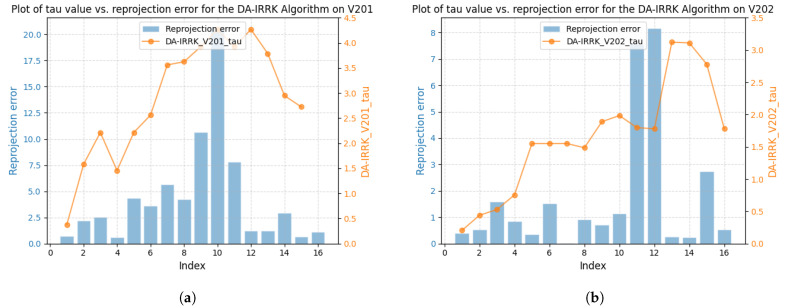
Relationship between reprojection errors and robustness parameter τ using the DA-IRRK method on sequences V201 and V202. (**a**) The left is for V201. (**b**) The right is for V202.

**Figure 14:**
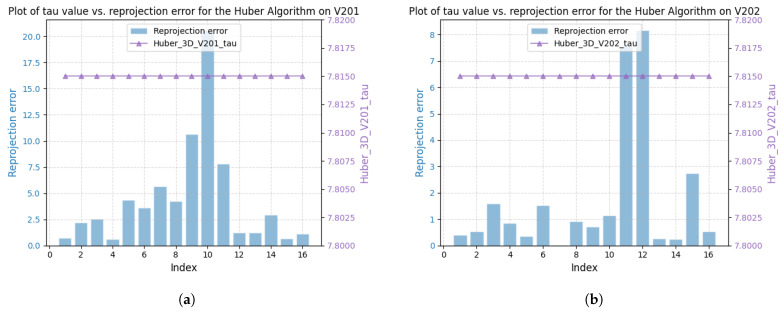
Relationship between reprojection errors and robustness parameter τ using the robust Huber method on sequences V201 and V202. (**a**) The left is for V201. (**b**) The right is for V202.

To analyze the reasons for the degradation of mapping accuracy on sequences V201 and V203, we conducted a comparative analysis using sequences V201 and V202 as examples. In accordance with Section 4.3.1, our analysis begins with the proposed DA-IRRK method. Figure 13 and Figure 14 show the relationship between reprojection error and robustness parameters for both methods on sequences V201 and V202. The results consolidate those in the visual-only SLAM case.

#### 4.3.3. Collaborative VSLAM

In this subsection, the effectiveness of the DA-IRRK approach is tested in collaborative VSLAM frameworks such as JORB-SLAM and CCM-SLAM, with only two agents adopted. Specifically, as in [7,16], we also test the proposed method in JORB-SLAM on the KITTI dataset and CCM-SLAM on the EuRoC dataset. JORB-SLAM divides a trajectory segment into overlapping parts in its experiments to achieve mapping through multi-robot collaboration, while CCM-SLAM achieves mapping by multiple robots collaborating to run respective maps.

In the experiments with CCM-SLAM, sequences from EuRoC are paired together (e.g., MH01-MH02, where agent A runs MH01 and agent B runs MH02) with map fusion performed at a central station. Evaluation is based on the RMSE of ATE compared only with the most widely used robust Huber kernel to avoid redundancy. Results are detailed in Table 4 and Table 5. The third row, Impr_huber, in Table 4 and Table 5, quantifies the improvement of the proposed method compared with the Huber function-based method.

The rule is developed to evaluate accuracy within a collaborative framework: the accuracy of the final trajectory is considered improved if both agent A and agent B enhance their trajectory accuracy after running on the dataset; additionally, the accuracy of the final trajectory is also considered enhanced if only one agent (A or B) improves its trajectory accuracy after running on the dataset but with the improvement higher than the accuracy degradation of the other; conversely, in any other case, we consider the accuracy of the final trajectory decreased. Following the above stipulation, we can see from Table 4 that the mapping accuracy on sequences 04, 07, and 08 decreases, while the mapping accuracy on the other 7 sequences improves greatly, with sequences 01, 02, 03, 06, and 10 improving by more than 10.23%.

**Figure 15:**
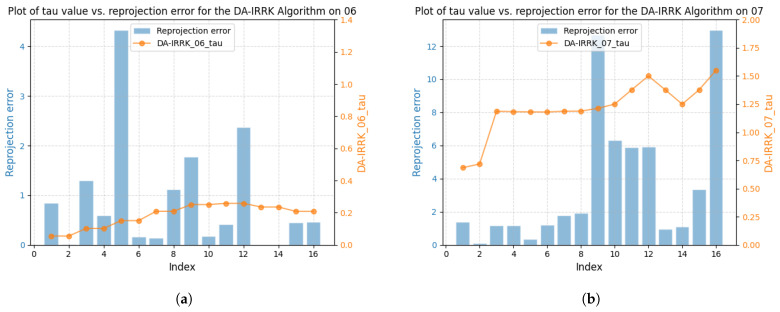
Relationship between the reprojection error and the robustness parameter τ using the DA-IRRK method on sequences 06 and 07. (**a**) The left is for 06. (**b**) The right is for 07.

**Figure 16:**
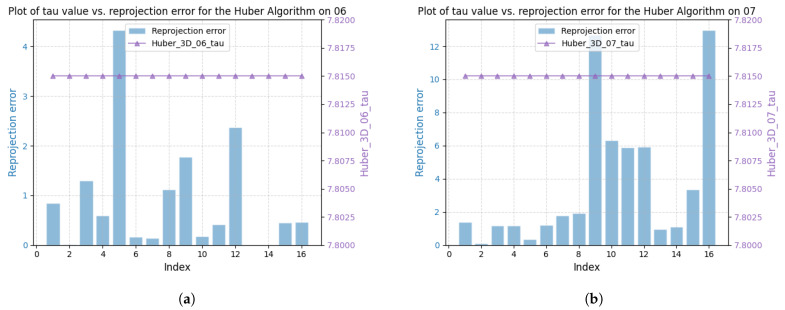
Relationship between the reprojection error and the robustness parameter τ using the robust Huber method on sequences 06 and 07. (**a**) The left is for 06. (**b**) The right is for 07.

**Figure 17:**
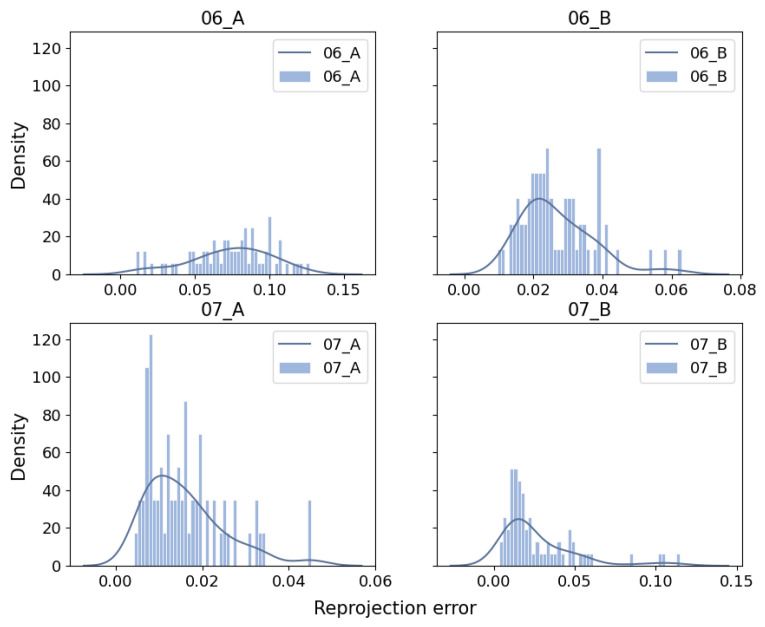
Reprojection error distribution of sequences 06 and 07 for local mapping in the JORB-SLAM framework.

We take sequences 07 and 06 as an example to demonstrate the performance difference. From Figure 15 and Figure 16, the robustness parameter of the DA-IRRK method fluctuates with the reprojection error, whereas that of the robust Huber kernel method remains constant. We can also see that the robustness parameter of sequence 06 is smaller than that of sequence 07, and similarly, the reprojection error of sequence 06 is smaller than that of sequence 07, which demonstrates that sequence 06 has a better mapping accuracy, which is consistent with the results in Table 4. In the reprojection error analysis of the sub-sequences of sequences 06 and 07, as shown in Figure 17. It is worth noting that sequence 06 tends to be a sub-Gaussian distribution, while sequence 07 tends to be a non-Gaussian distribution, and according to [14], we can know that the proposed DA-IRRK method is more effective in suppressing the sub-Gaussian distribution of error data, and consequently, the mapping accuracy of sequence 06 is better.

**Figure 18:**
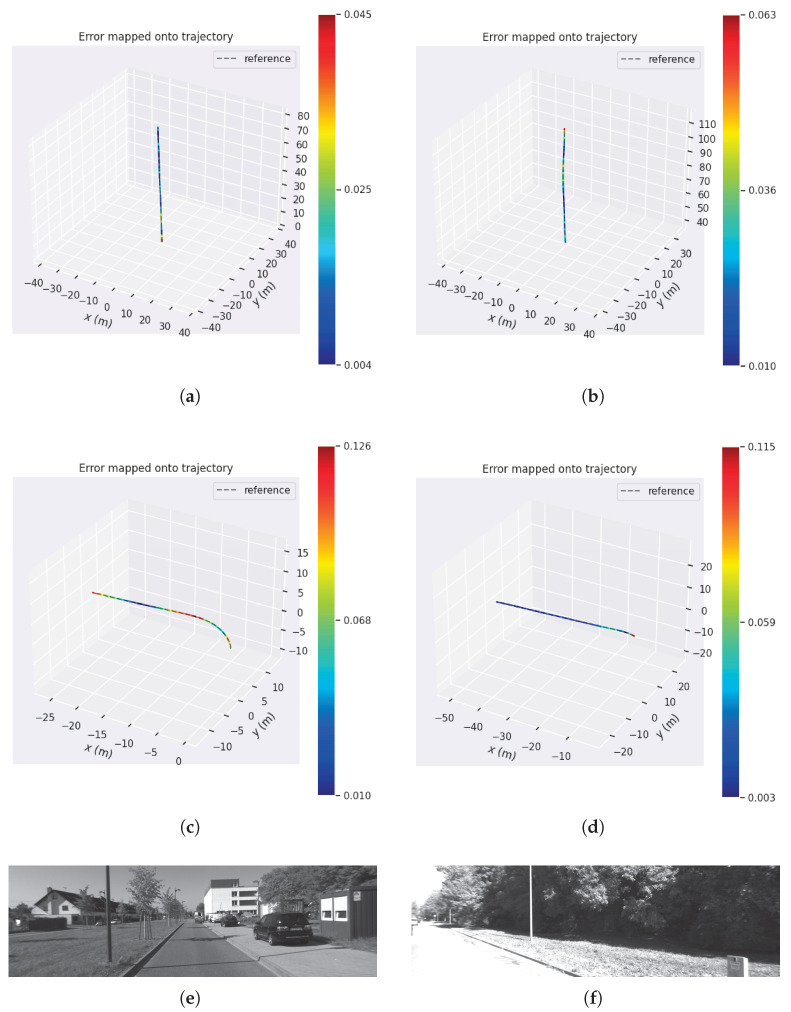
Scene and trajectory on sequences 06 and 07 in the JORB-SLAM framework. (**a**,**b**) Sequence 06 trajectory, (**c**,**d**) Sequence 07 trajectory, (**e**) Sequence 06 scene, (**f**) Sequence 07 scene.

The reprojection error is related to the actual environment, and different environments have different reprojection error distributions. As can be seen from Figure 18, the trajectory of sequence 06 is relatively simple, characterized by numerous trees and moderate lighting, which provides clearer feature points for optimal tracking. On the other hand, the scene of sequence 07 has strong lighting, resulting in unclear feature points and, consequently, more mismatches during tracking, thereby reducing mapping accuracy.

The experimental results in the CCM-SLAM framework are presented in Table 5. We can see a significant accuracy difference between the two agents on MH03-MH04 and MH04-MH05, with one agent showing a much larger improvement than the other. Table 5 reveals that the DA-IRRK method outperforms the robust Huber kernel method in terms of mapping accuracy within the CCM-SLAM framework. This unexpected result opens up new possibilities for future advancements in VSLAM collaboration by improving accuracy levels.

Summarizing the above analysis, we can conclude the proposed DA-IRRK method not only works properly in different visual collaborative SLAM frameworks but also shows better mapping accuracy, which proves the effectiveness as well as the robustness of the proposed method in collaborative VSLAM frameworks.

The experiments in Section 4.3 demonstrate that while the DA-IRRK method improves mapping accuracy over the robust Huber kernel, this comes with an increased computational cost. To evaluate real-time performance, we conducted timing analysis on the EuRoC V103 sequence using ORB-SLAM3 (Table 6, where the top performer is bolded.). Results show the DA-IRRK method maintains real-time capability (≤33 ms/frame at 30 Hz) in both visual-only and visual-inertial modes, with only marginal time increases (≤0.1 s) versus the Huber kernel. The visual-inertial mode’s reduced keyframe optimization further minimizes computational overhead. Our frame-rate compliance tests confirm the method’s practical suitability for real-time VSLAM applications.

## 5. Conclusions

In VSLAM, the robust kernel method is commonly used for back-end optimization, but its fixed robustness parameter limits performance in varying scenarios. To address this, we developed a data-driven, adaptive, iteratively reweighted robust kernel method to improve accuracy and robustness. Experiments in both indoor and outdoor environments show that our method outperforms robust Huber, Cauchy, and Tukey kernels, as well as state-of-the-art MCMCC methods, particularly when sub-Gaussian distributions occur during reprojection. Additionally, multi-sensor experiments reveal that our method surpasses the robust Huber kernel, with lower performance only in sequences with large reprojection errors. Furthermore, in the JORB-SLAM and CCM-SLAM frameworks, our method not only operates efficiently but also achieves higher accuracy. However, it shares a limitation with the robust Huber kernel in non-Gaussian environments, where it underperforms compared with the latest MCMCC method. Future work will focus on (1) developing data-adaptive methods for non-Gaussian features in VSLAM; (2) extending the approach to multi-modal sensor fusion applications; and (3) optimizing real-time performance for edge computing devices.

## Figures and Tables

**Table 1:** RMSE ATE (m) on Stereo EuRoC in ORB-SLAM3.

	MH01	MH02	MH03	MH04	MH05	V101	V102	V103	V201	V202	V203
Ours	**0.0363**	**0.0308**	0.0405	**0.0601**	**0.0461**	**0.0788**	**0.0603**	0.0738	0.0748	**0.0557**	0.5490
Huber	0.0391	0.0333	0.0411	0.0639	0.0529	0.0826	0.0637	0.0749	0.0729	0.0582	0.4940
MCMCC	0.0380	0.0332	**0.0373**	0.0660	0.0518	0.0826	0.0641	**0.0729**	**0.0528**	0.0565	**0.4150**
**Cauchy**	0.0395	0.0332	0.0442	0.0814	0.0534	0.0809	0.0672	0.1160	0.0744	0.0683	0.8560
Tukey	0.0391	0.0345	0.0433	0.0892	0.0518	0.0815	0.0647	0.1140	0.0832	0.0859	0.8410

**Table 2:** RMSE ATE (m) on Stereo KITTI in ORB-SLAM3.

	00	01	02	03	04	05	06	07	08	09	10
Ours	**0.9445**	**5.0316**	**5.2060**	0.2806	**0.1794**	**0.3591**	0.6449	**0.4207**	**2.7705**	1.7737	0.9458
Huber	0.9454	5.8984	6.1795	0.2962	0.1990	0.3920	0.6357	0.4833	2.8597	**1.7117**	1.0910
MCMCC	0.9567	5.6801	5.5742	**0.2309**	0.1978	0.3708	0.6309	0.4224	2.9752	1.7946	**0.9226**
Cauchy	1.0448	9.1711	5.8003	0.2945	0.1833	0.3958	0.7584	0.7923	3.4113	1.7704	1.0993
Tukey	0.9932	7.9178	7.5824	0.3973	0.2941	0.4572	**0.5805**	0.5024	2.8851	1.8602	1.6109

**Table 3:** Multi-sensor combination (visual-inertial): RMSE ATE (m) for Stereo-mode testing using ORB-SLAM3 on EuRoC dataset (indoor).

	MH01	MH02	MH03	MH04	MH05	V101	V102	V103	V201	V202	V203
Ours	**0.0190**	**0.0154**	**0.0228**	**0.0408**	**0.0535**	**0.0326**	**0.0075**	**0.0161**	0.0147	**0.0102**	0.0164
Huber	0.0231	0.0209	0.0258	0.0454	0.0560	0.0338	0.0087	0.0171	**0.0135**	0.0119	**0.0153**
Impr_huber	18.08%	26.29%	11.92%	10.02%	4.64%	3.64%	13.53%	6.13%	−9.44%	14.68%	−6.60%

**Table 4:** RMSE ATE (m) on Stereo-KITTI in JORB-SLAM.

		00	01	02	03	04	05	06	07	08	09	10
Ours	A	0.1845	0.1128	0.0590	0.0664	0.0648	0.0564	0.0184	0.0785	1.0785	0.0449	0.0397
	B	0.1023	0.3891	0.0849	0.0441	0.0662	0.0512	0.0288	0.0358	0.4829	0.0643	0.0397
Huber	A	0.1881	0.1591	0.0828	0.0657	0.0614	0.0625	0.0226	0.0754	1.0793	0.0519	0.0442
	B	0.1093	0.4829	0.0973	0.0524	0.0645	0.0537	0.0335	0.0322	0.4822	0.0677	0.0558
Impr_huber	A	1.91%	29.10%	28.73%	−0.94%	−5.49%	9.83%	18.45%	−4.12%	0.07%	13.52%	10.23%
	B	6.40%	19.42%	12.82%	15.84%	−2.64%	4.65%	13.87%	−11.12%	−0.15%	5.02%	28.88%

**Table 5:** RMSE ATE (m) on Mono-EuRoC in CCM-SLAM.

	MH01-MH02	MH02-MH03	MH03-MH04	MH04-MH05	V101-V102	V102-V103	V201-V202	V202-V203
Ours	MH01 0.1296	MH02 0.1202	MH03 0.1506	MH04 0.1710	V101 0.0093	V102 0.1046	V201 0.0290	-
	MH02 0.1066	MH03 0.1077	MH04 0.1222	MH05 0.1626	V102 0.0347	V103 0.1025	V202 0.03576	-
Huber	MH01 0.3069	MH02 0.1862	MH03 0.2049	MH04 1.0253	V101 0.1261	V102 0.1525	V201 0.0860	-
	MH02 0.2508	MH03 0.1397	MH04 0.1186	MH05 0.1620	V102 0.1464	V103 0.2125	V202 0.0988	-
Impr_huber	MH01 57.77%	MH02 35.45%	MH03 26.50%	MH04 83.32%	V101 92.62%	V102 31.41%	V201 66.28%	-
	MH02 57.50%	MH03 22.91%	MH04 −3.04%	MH05 −0.37%	V102 76.30%	V103 51.76%	V202 59.21%	-

**Table 6:** Computational elapsed time of the main modules on V103 of EuRoC in ORB-SLAM3 framework (meantime and standard deviation in ms).

	Al	Huber	DA-IRRK (Ours)	Huber	DA-IRRK (Ours)
Settings	Sensor	Stereo	Stereo	Mono-Inertial	Mono-Inertial
	Cam.Resolution	752 × 480	752 × 480	600 × 350	600 × 350
	Cam.fps	20 Hz	20 Hz	20 Hz	20 Hz
	IMU	-	-	200 Hz	200 Hz
	ORB Feat.	1200	1200	1000	1000
	RMSE ATE	0.08269	**0.06968**	0.03729	**0.01611**
Tracking	ORB extract	14.566	15.259	6.137	5.941
	Stereo match	2.7792	2.7234	-	-
	IMU integr.	-	-	0.0478	0.04515
	Pose pred	1.633	2.243	0.051	0.044
	Local Mapping (LM) Track	2.918	3.703	4.491	4.399
	New Key Frame (KF) dec	0.0715	0.0762	0.05	0.0463
	Total	**24.416**	26.583	-	-
Local Mapping (LM)	KF Insert	3.879	4.064	6.337	6.317
	Map Point (MP) Culling	0.254	0.291	0.0743	0.0773
	MP Creation	10.051	10.269	20.797	20.591
	**Local BA (LBA)**	**38.444**	60.007	63.51	**62.972**
	KF Culling	3.489	3.379	17.834	16.637
	Total	**55.655**	77.275	107.93	**107.26**
LBA complexity	LBA Edges	9087.7	**9203**	1628.3	**1727.6**
	LBA KF optimized	21.067	**20.584**	5.923	5.833
	LBA KF fixed	37.366	35.174	1	1
	Map Size KFs	165	160	341	346
	Map Size MPs	7979	8348	11572	11308
Full BA	**Global BA (GBA)**	**130.73**	230.62	775.59	**686.97**
	Map Update	16.793	4.1786	57.345	40.312
	Total	**147.52**	234.8	832.94	**727.28**
	BA Size KFs	73	73	139	126
	BA Size MPs	5020	5108	6330	5406

## Data Availability

The dataset used in the paper is the public KITTI raw data, which can be downloaded at https://www.cvlibs.net/datasets/kitti/raw_data.php, (accessed on 1 December 2024). The other dataset used in the paper is the public EuRoC Dataset, which can be downloaded at https://projects.asl.ethz.ch/datasets/doku.php?id=kmavvisualinertialdatasets, (accessed on 1 December 2024).
